# *In Vitro* Antioxidant and Antibacterial Activities of Medicinal Flower Laligurans *Rhododendron arboreum* Collected from Kathmandu Valley, Nepal

**DOI:** 10.1155/2024/6073042

**Published:** 2024-07-16

**Authors:** Anil Kumar Jha, Monowar Alam Khalid, Shyam Narayan Labh

**Affiliations:** ^1^ Department of Environment Science Integral University, Kursi Road 226026, Lucknow, Uttar Pradesh, India; ^2^ Aquaculture Research Unit Amrit Science Campus Tribhuvan University, Kathmandu 44600, Nepal

## Abstract

*Rhododendron arboreum*, known as Nepal's national flower and belonging to the Ericaceae family, thrives throughout the Himalayan region. This study investigates the antioxidant and antimicrobial potential of *Rhododendron arboreum* flowers. Three different extracts were prepared at concentrations ranging from 10 to 640 *μ*g/mL and assessed for their total phenolic content (TPC), total flavonoid content (TFC), and DPPH radical scavenging activity. Results showed significant accumulation of antioxidant compounds (*P* < 0.05), with TPC levels of 37.78, 67.29, and 53.46 mg GAE/g and TFC values of 49.46, 67.46, and 65.71 mg QE/g for LGP, LGE, and LGA at 640 *μ*g/mL, respectively. DPPH activity was highest in LGE (96.33%), followed by LGA (87.11%) and LGP (76.59%), compared to the standard (77.38%). The antibacterial properties were significant against *Aeromonas hydrophila*, *Staphylococcus aureus*, and *Escherichia coli* at 100% concentration, with inhibition rates of 15%, 16%, and 17% for LGP, LGE, and LGA, respectively. These findings indicate that *Rhododendron arboreum* petals, rich in bioactive compounds, possess strong antioxidant and antibacterial properties, making them potential candidates for developing cost-effective therapeutic formulations. Further research should focus on isolating specific bioactive compounds and understanding their molecular mechanisms to enhance therapeutic efficacy.

## 1. Introduction

Nepal, situated amid the geographic expanse connecting China and India, stands as a bastion of extraordinary biological diversity. Within the taxonomic realm of flora, the Ericaceae family encapsulates the botanical marvel known as *Rhododendron arboreum*, colloquially referred to as Laligurans. This species, endemic to Nepal and distinguished as the nation's floral emblem, thrives predominantly at elevations spanning from 1200 to 4000 meters above sea level [1]. *R. arboreum* floral composition has been noted to harbor quercetin-3-rhamnoside—a crystalline chemical composite comprising phenolic constituents, including quercetin, rutin, and coumaric acid, renowned for their potent physiological activity [2]. Noteworthy within the realm of botanical pharmacology, plant flowers serve as a cornerstone in the provision of medicinal resources, with approximately 80% of the global populace relying on plant-derived therapeutics as their primary healthcare recourse, owing to their intrinsic safety profile [3]. *R. arboreum*, in particular, holds therapeutic significance, finding utility in the management of diverse maladies encompassing febrile conditions, gastrointestinal afflictions, dermatological anomalies, hepatic inflammations, and the augmentation of nonspecific immune responses [4].

Medicinal flowers exhibit a remarkable capacity to combat microbial diseases owing to their biocompatibility and potential as reservoirs of novel antibacterial compounds [5]. Plant flowers have been demonstrated to harbor diverse bioactive constituents, including phenols, saponins, tannins, coumarins, ascorbic acid, flavonoids, terpenoids, anthraquinones, and glycosides [6]. Notably, approximately 80% of individuals in developing nations rely on traditional botanical remedies, underscoring the global significance of plant-derived therapeutics. The natural diversity of *Rhododendron* species surpasses 1,000 globally, with Nepal documenting the presence of around thirty *Rhododendron* varieties [7]. The burgeoning interest in plant extracts within the food industry is attributable to their antioxidant properties and associated health benefits, including their role in enhancing fish production in aquaculture [8]. Antioxidants function as vital protective agents against free radicals, thereby mitigating the risk of pathological conditions [9]. Flowers emerge as invaluable repositories of natural antioxidants, with extensive scientific inquiry attesting to their pivotal role in counteracting the deleterious effects of free radicals on pathological states [10]. In line with this perspective, a study was undertaken to examine the in vitro antioxidant and antibacterial properties of Laligurans *Rhododendron arboreum*, a medicinal flower sourced from the Kathmandu Valley in Nepal. This investigation is aimed at exploring the potential of these flower extracts as supplementary sources of antioxidants to enhance animal health.

## 2. Materials and Methods

### 2.1. Material Required

Ethanol, acetone, Folin-Ciocalteu's phenol reagent, gallic acid, sodium carbonate, sodium nitrite, aluminum chloride, sodium hydroxide, catechin, 2,2-diphenyl-1-picrylhydrazyl (DPPH), methanol, and ascorbic acid were procured from Sigma-Aldrich Chemicals Pvt. Ltd., India.

### 2.2. Taxonomic Identification

*Rhododendron arboretum* (Laligurans) was collected from Phulchowki in the Kathmandu Valley. For taxonomic identification, the obtained flower samples were double sealed in a plastic bag, shade dried, and sent to the National Herbarium and Plant Laboratories in Godavari, Kathmandu.

### 2.3. Extraction Mechanism of Flower

**Figure 1:**
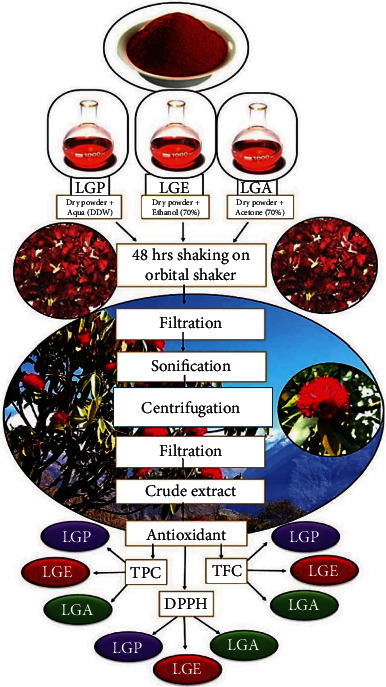
Extraction mechanism from the flower petals of *Rhododendron arboreum.*

The extraction procedures (Figure 1) employed in this study were a combination of methods described by [11, 12] and Labh et al. [13]. The flower petals of *R. arboreum* underwent a ten-day air-drying process at room temperature, followed by pulverization in a blender and storage in a container. Three distinct crude extract solutions were prepared using different techniques. In sample 1 (LGP), 20 g of dried Laligurans powder was mixed with 200 mL of distilled deionized water (DDW), while in sample 2 (LGE), 20 g of dried Laligurans powder was mixed with 200 mL of 70% ethanol, and in sample 3 (LGA), 20 g of Laligurans powder was dissolved in 200 mL of 70% acetone. Each sample was sealed within cotton plugs and aluminum foil before undergoing agitation in an orbital shaker with a magnetic stirrer for 48 hours. The resulting crude extracts (LGP, LGE, and LGA) were stored in corked round bottom flasks. Centrifugation at 10,000 revolutions per minute for 5 minutes facilitated the separation of solids, after which the liquid supernatant was filtered using Whatman Filter Paper Grade No. 1—size 110 mm. The solvent was then evaporated using nitrogen gas at room temperature and stored at 4°C for future utilization. Assessment of antioxidant capabilities involved measuring the total phenolic content (TPC) and total flavonoid content (TFC) and employing the novel 2,2-diphenyl-1-picrylhydrazyl method. Additionally, the antibacterial activity of the flower extracts was assessed against three bacterial strains: *Aeromonas hydrophila*, *Staphylococcus aureus*, and *Escherichia coli*.

### 2.4. Total Phenolic Content (TPC)

The Folin-Ciocalteu procedure [14], with modifications [15], was employed to quantify the total phenolic content. Specifically, 0.1 mL of crude extracts (LGP, LGE, and LGA) was mixed with 3 mL of distilled water and subsequently treated with 500 *μ*L of 2N Folin-Ciocalteu phenol reagent. Following an incubation at 28°C for 3 minutes, 2 mL of sodium carbonate solution (20% *w*/*v*) was added. The resulting mixture was incubated in the dark for one hour. Absorbance readings for each sample were taken at 760 nm using a UV-1800 spectrophotometer (Shimadzu Corp., Kyoto, Japan). A calibration curve was established using various concentrations of a gallic acid solution (500 mg/L), yielding a standard curve equation of *y* = 0.041*x* + 0.022 with an *R*^2^ value of 0.979. The total phenolic content was expressed as milligrams of gallic acid equivalents per gram of dry weight (mg GAE/g DW).

### 2.5. Total Flavonoid Content (TFC)

The total flavonoid content (TFC) of the extracts (LGP, LGE, and LGA) was quantified using the colorimetric method described by Babotă et al. [16]. 3 mL of diluted extract was mixed with 4 mL of distilled water and 300 *μ*L of 5% (*w*/*v*) NaNO_2_ in a 15 mL conical glass tube. The solution was incubated for 5 minutes, followed by the addition of 300 *μ*L of 10% (*w*/*v*) AlCl_3_. After a 6-minute incubation period, 2 mL of a 1 M sodium hydroxide solution was added. The final volume was adjusted to 10 mL with deionized water. The mixture was incubated at 28°C for 15 minutes, resulting in a pink color change. The absorbance was measured at 510 nm against a blank sample. The TFC was determined using a calibration curve with the standard equation *y* = 0.001*x* − 0.002 and an *R*^2^ value of 0.997, established with various concentrations of catechin (20-100 *μ*g). Results are expressed as milligrams of catechin equivalent per gram of dry weight (mg CE/g DW).

### 2.6. DPPH (2,2-Diphenyl-1-picrylhydrazyl) Free Radical Assay

The DPPH radical scavenging activity of the extracts was assessed according to the method described by [17], with slight modifications based on the study by Labh et al. [18]. A 0.05-millimolar (mM) solution of 2,2-diphenyl-1-picrylhydrazyl (DPPH) was prepared in methanol at a low temperature. Subsequently, 200 *μ*L of this DPPH solution was added to each reaction mixture, which consisted of 3.8 milliliters (mL) of methanol and a sample extract ranging from 50 to 250 *μ*L. Ascorbic acid (vitamin C) was used as a positive control in the experiment. The mixtures were incubated in a dark environment at 37°C for 30 minutes. After incubation, the absorbance was measured at a wavelength of 517 nm. The DPPH radical scavenging activity was quantified by calculating the percentage using the following equation: DPPH scavenging activity (%) = (*A*_0_ − *A*_1_)/*A*_1_ × 100, where *A*_0_ is the absorbance of the control and *A*_1_ is the absorbance of the sample.

### 2.7. Antimicrobial Properties of the Floral Extracts

#### 2.7.1. Bacterial Strains

The antimicrobial activity of three extracts (LGP, LGE, and LGA) obtained from the flowers of *Rhododendron arboreum* was evaluated against two Gram-negative bacteria (*Aeromonas hydrophila* and *Escherichia coli*) and one Gram-positive bacterium (*Staphylococcus aureus*), all of which are known to cause foodborne illnesses. The bacterial strains were acquired from the Fish Disease Diagnostic Laboratory, Nepal Agriculture Research Council (NARC), Nepal.

#### 2.7.2. Inoculum Preparation

Utilizing stock solutions, bacterial cultures were propagated in nutrient broth for a 22-hour incubation period under controlled conditions at 25°C [19]. Subsequently, the optical density of bacterial growth was assessed at 580 nm employing a spectrophotometer. Following this, the sample underwent dilution to attain a viable cell count of 10^7^ CFU/mL. A 5 mL aliquot of sterile saline solution was employed for the collection of bacterial growth. The experimental setup involved the utilization of 100 mL volumes of unrefined extract at four distinct concentrations: 25%, 50%, 75%, and 100%. Each assay disc was immersed in its respective extract for a 24-hour duration at a controlled temperature of 37°C, following which the degree of inhibition was quantified in millimeters (mm). Importantly, the experimental procedure was replicated in triplicate to ensure the reliability and reproducibility of results.

#### 2.7.3. Flower Extract's Ability to Kill Germs

The antimicrobial efficacy of the three extracts was assessed using the disc diffusion method against *Aeromonas hydrophila*, *Staphylococcus aureu*s, and *Escherichia coli.* To prepare the agar plates, 10 mL of Mueller-Hilton agar medium and 15 mL of previously inoculated seeded media (containing 100 mL of medium per mL with a concentration of 10^5^ CFU/mL) were added to sterile Petri dishes. Sterile filter paper discs, coated with plant extract at a concentration of 10 mg/mL, were placed onto the Mueller-Hilton agar plates. Additionally, filter paper discs impregnated with 5 g of gentamycin served as positive controls. Following placement of the discs, the plates were left at room temperature for 24 hours to allow for the diffusion of plant extracts. Subsequently, the antibacterial activity was assessed by measuring, recording, and interpreting the presence of inhibitory zones using Vernier calipers.

### 2.8. Statistical Analysis

An analysis of variance (ANOVA) and Duncan's multiple range tests (DMRTs) were conducted for the data in this study using SPSS (version 20) at a significance level of *P* < 0.05. The data is reported as the average value plus or minus the standard deviation of three repeated tests.

## 3. Results

### 3.1. *In Vitro* Antioxidant Assays of *R. arboreum*

#### 3.1.1. Total Phenolic Content (TPC)

**Figure 2:**
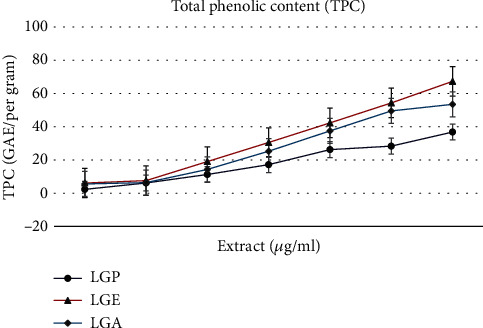
Total phenolic content (TPC) of different extracts of *R. arboreum* flowers.

The study examined the results of total phenolic content (TPC) as shown in Figure 2. A statistically significant (*P* < 0.05) increase in TPC was observed in the flower extracts. The TPC content of the extracts showed a linear trend, increasing with the concentration of the extract. LGE exhibited the highest total phenolic content, containing 45% and 20% more TPC compared to LGP and LGA, respectively, at a concentration of 640 *μ*g/mL. The highest TPC accumulation at this concentration was 36.78 ± 0.31 mg gallic acid equivalent (GAE) per gram for LGP, 67.29 ± 0.33 mg GAE/g for LGE, and 53.46 ± 0.23 mg GAE/g for LGA. At the concentration of 10 *μ*g/mL, the lowest observed TPC accumulation was 2.29 ± 0.09 mg GAE/g, 6.10 ± 0.13 mg GAE/g, and 5.55 ± 0.44 mg GAE/g dry weight in LGP, LGE, and LGA, respectively. The TPC was found to be maximum at a concentration of 640 *μ*g/mL in all three extracts.

#### 3.1.2. Total Flavonoid Content (TFC)

**Figure 3:**
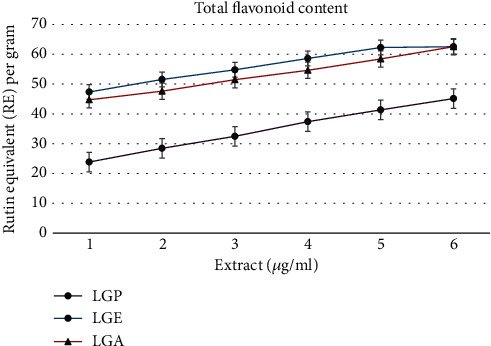
Total flavonoid content (TFC) of different extracts of *R. arboretum* flowers.

The total flavonoid content (TFC) in the extracts of *R. arboreum* flowers is presented in Figure 3. A similar trend was observed for TFC as with TPC, showing an increase with the concentration of the extract. The buildup of TFC in LGE was approximately twice as high as in the other samples. Like TPC, TFC was significantly higher (*P* < 0.05) at a concentration of 640 *μ*g/mL in all three extracts (LGP, LGE, and LGA). The highest TFC values were 49.46 ± 0.21 mg rutin equivalent (RE) per gram dry weight (DW) for LGP, 67.46 ± 0.17 mg RE/g DW for LGE, and 65.71 ± 0.17 mg RE/g DW for LGA. The lowest TFC recorded was 23.85 ± 0.32 mg RE/g DW in LGP, 47.33 ± 0.23 mg RE/g DW in LGE, and 44.71 ± 0.15 mg RE/g DW in LGA.

#### 3.1.3. DPPH Radical Scavenging Activity

The efficacy of the LGP, LGE, and LGA extracts in scavenging the DPPH radical was quantified as a percentage of inhibition and compared to ascorbic acid (AA) as a reference (Table 1). The lowest level of DPPH inhibition was observed at a concentration of 10 *μ*g/mL (Table 1). The IC50 values for LGP, LGE, and LGA were higher than the IC50 value for ascorbic acid, which was 738 *μ*g/mL. Specifically, the IC50 values for LGP, LGE, and LGA were 646 *μ*g/mL, 926 *μ*g/mL, and 817.11 *μ*g/mL, respectively. As shown in Table 1, the flower extracts LGP, LGE, and LGA exhibited higher percentage inhibition of the DPPH radical at a concentration of 640 *μ*g/mL compared to the ascorbic acid standard (77.38%). Specifically, LGP, LGE, and LGA showed inhibition rates of 76.59%, 96.33%, and 87.11%, respectively.

### 3.2. Antibacterial Activity of Flower Extracts

The unrefined extracts at concentrations of 25%, 50%, 75%, and 100% obtained from the flower petals of *Rhododendron arboreum* were used to assess antibacterial activity against bacterial strains. The antibacterial effectiveness of three extracts, specifically LGP, LGE, and LGA, was evaluated against two Gram-negative bacterial strains (*Aeromonas hydrophila* and *Escherichia coli*) and one Gram-positive bacterium (*Staphylococcus aureus*) associated with foodborne illness.

The results indicated that all three extracts possess antibacterial properties and can be used as effective treatments against these food poisoning bacteria (Table 2). The least significant antibacterial effect was observed in *Aeromonas hydrophila* (1.37 ± 0.56 mm inhibition zone), followed by *Staphylococcus aureus* (1.76 ± 0.19 mm) and *Escherichia coli* (1.91 ± 0.11 mm) when subjected to LGP and LGE extracts at a concentration of 25%. The antibacterial properties intensified with higher concentrations of the extracts. The maximum recorded inhibition zones were 21.41 ± 0.63 mm in *Staphylococcus aureus* for the LGE extract, 20.13 ± 0.93 mm in *Escherichia coli* for the LGA extract, and 18.28 ± 0.94 mm in *Aeromonas hydrophila* for the LGE extract.

## 4. Discussion

Herbal medicine holds a significant position within the realm of traditional medicine [20]. Over the past twenty years, there has been a substantial increase in the interest in investigating natural substances as potential sources of innovative antibacterial drugs. This study examined various extracts derived from traditional medicinal herbs to identify the origins of their therapeutic benefits [21–24]. To promote the appropriate utilization of herbal medicines and explore their potential as new pharmaceutical sources, this study investigated the potential of *Rhododendron arboreum* flower petals, native to Nepal, in inhibiting oxidative damage by analyzing their antioxidant content, including total phenolic content (TPC), total flavonoid content (TFC), and DPPH radical activity.

Consequently, specific natural compounds have obtained FDA approval as novel antibacterial drugs. Nevertheless, the need to discover new compounds effective against diseases with strong resistance remains crucial. Over numerous centuries, a wealth of knowledge regarding the healing properties of plants has been accumulated. The local community possesses extensive traditional knowledge regarding the use of various plants or substances for treating common diseases [25]. According to research, total phenolic content (TPC) is considered the primary compound in plants capable of inhibiting free radicals. In this study, the total phenolic content of LGP, LGE, and LGA extracts was measured at a concentration of 10 *μ*g/mL, with results showing 2.44% for LGP, 6.11% for LGE, and 5.61% for LGA. These values increased significantly at a higher concentration of 640 *μ*g/mL, reaching 37.06% for LGP, 67.74% for LGE, and 53.83% for LGA. Notably, at this concentration level, LGE exhibited an 11.37% greater TPC compared to the other samples (Figure 2).

Flavonoids are naturally present in fruits, vegetables, nuts, and tea. They are a type of polyphenolic compound with diverse chemical structures and characteristics. Flavonoids are primarily recognized for their potent antibacterial effects against a wide range of microorganisms. Their ability to bind with proteins outside of cells, soluble proteins, and bacterial cell walls is associated with their antibacterial function. Multiple investigations have documented the antibacterial efficacy of various botanical extracts. These studies confirm prior research suggesting that tannins derived from *Rhododendron arboreum* flowers possess exceptional antibacterial properties and may hold medicinal importance. Additionally, these extracts exhibit both antioxidant and anti-inflammatory characteristics. In this study, the total flavonoid content (TFC) of the LGP, LGE, and LGA extracts was measured. At a concentration of 10 *μ*g/mL, the TFC percentages were 24.23%, 47.66%, and 44.06%, respectively. At a higher concentration of 640 *μ*g/mL, the TFC percentages increased significantly, with LGE and LGA showing an 8.67% and 7.88% increase compared to LGP, respectively (Figure 3). TFC is present in various constituents of all three extracts.

The DPPH free radical scavenging activity was evaluated using various concentrations (ranging from 10 to 640 *μ*g/mL) of LGP, LGE, and LGA extracts derived from the petal parts of *Rhododendron* flowers. These flower extracts exhibited the greatest antioxidant capacity compared to the other sections analyzed (Table 1). The results of this experiment demonstrated that higher concentrations of the extracts resulted in increased activity in removing free radicals. Notably, the floral extracts demonstrated the highest DPPH scavenging activity at a concentration of 640 *μ*g/mL, with 76.59 ± 0.13% activity in LGP, 96.33 ± 0.37% activity in LGE, and 87.1% activity in LGA. In comparison, AA (ascorbic acid) showed 77.38 ± 0.6% activity, making it 24.47% lower than the LGE standard. The IC50 concentrations for LGP, LGE, LGA, and AA were 646.59, 926.33, 817.11, and 738.12 *μ*g/mL, respectively. However, the significance of the values of all three extracts compared to the standard was extremely significant (*P* < 0.05) at all levels of DPPH radical activity concentration (Table 1).

Consistent with the current observations, various flowers have been tested for their antibacterial properties against prevalent and harmful bacteria [26]. In their study, Gokul et al. [27] discovered that the flower extract of *Catharanthus roseus* exhibited wound-healing properties in fish. Karnwal [28] conducted a study to explore the antibacterial capabilities of *N. indicum* and *H. rosa-sinensis* on ten distinct kinds of Gram-negative bacteria. Additionally, Park et al. [29] evaluated the antimicrobial efficiency of traditional flower vegetable extracts. In their study, Manandhar et al. [30] examined the antibacterial efficacy of crude methanolic extracts derived from various sections of the plant against pathogenic bacteria. Furthermore, the antibacterial activity of various plants was evaluated by testing their varied solvent extractions against a range of harmful bacteria. It was found that flowers were particularly beneficial in their practical uses [31–34].

The present investigation employed three bacterial strains (*Aeromonas hydrophila*, *Staphylococcus aureus*, and *Escherichia coli*) to evaluate the antibacterial efficacy of floral extracts (Table 2). A direct association between the extract concentration and its antibacterial potency was observed. Notably, when the concentration of LGE reached 100%, the antibacterial activity against *A. hydrophila*, *S. aureus*, and *E. coli* was measured at 18.28%, 21.41%, and 20.13%, respectively. Conversely, the effectiveness of LGP extracts against *A. hydrophila*, LGE extracts against *S. aureus*, and LGP extracts against *E. coli* was constrained at lower concentrations (25%), with corresponding values of 1.37, 1.76, and 1.91, respectively. In parallel, a study by Kashyap et al. [35] demonstrated that the ethanolic extract of *Rhododendron arboreum* exhibited superior efficacy in inhibiting Gram-negative bacteria compared to Gram-positive counterparts. The underlying mechanism proposed suggests that the ethanolic extract disrupts the lipopolysaccharide membrane of Gram-negative bacteria, leading to cellular destruction. However, conflicting findings were reported by Lal et al., wherein methanolic extracts exhibited greater effectiveness in inhibiting pathogenic growth compared to ethanolic counterparts [36].

*Aeromonas* poses a significant risk to the well-being of fish, especially in densely populated aquaculture systems that employ intense feeding practices. *Aeromonas hydrophila*, a single-flagellum bacterium commonly found in freshwater, primarily infects fish. *Staphylococcus aureus* also colonizes the skin and mucous membranes of food handlers during transportation, and its prevalence has been detected in fish from different countries. *Escherichia coli*, a Gram-negative bacillus, is commonly present in the intestines, muscles, skin, and gills of fish. Phenolic chemicals, including tannins, present in flower extracts are highly effective at killing microorganisms. Therefore, crude extracts LGP, LGE, and LGA may contain several organic components, such as flavonoids, tannins, alkaloids, and triterpenoids, all of which are known to have antibacterial properties. In summary, natural compounds must possess antibacterial properties. The antibacterial activity of the methanolic and acetone leaf extract of *Rhododendron arboreum* was investigated against various pathogens [37, 38].

## 5. Conclusions

*Rhododendron arboreum*, commonly known as Laligurans, holds prestigious recognition as the official tree of Sikkim and Uttarakhand in India, the designated flower of Washington in the United States, and the esteemed national flower of Nepal. Recognized for its robust antioxidant properties, this botanical specimen confers therapeutic advantages to human health. The flowers of *Rhododendron arboreum* possess versatile applications in the food and beverage industry and are esteemed for their manifold health benefits, including anti-infective properties. The present study delved into the antioxidant potential and antimicrobial activity of three distinct types of crude extract solutions, LGP (aqueous), LGE (ethanol 70%), and LGA (acetone 70%), aiming to elucidate their effects on total phenolic, total flavonoid, and DPPH radical activities. Notably, the ethanolic extract, primarily LGE, exhibited the highest antioxidant activity and antimicrobial efficacy against the studied bacteria: *A. hydrophila*, *S. aureus*, and *E. coli*. The observed antimicrobial activity of the extract is attributed to the presence of bioactive compounds inherent in the flower. However, the absence of activity in certain cases may be attributed to active chemical degradation occurring during the drying and extraction processes. Further, a comprehensive and sophisticated analysis is warranted to elucidate the specific compounds responsible for the antimicrobial effect and their potential mechanisms of action.

## Figures and Tables

**Table 1:** DPPH radical scavenging activity of different extract of *R. arboreum* flowers.

Concentration (*μ*g/mL)	DPPH radical activity			
	LGP	LGA	LGE	AA
10	6.59 ± 0.13^a^	16.33 ± 0.15^a^	17.10 ± 0.13^a^	5.38 ± 0.16^a^
20	17.10 ± 0.19^b^	20.48 ± 0.37^b^	19.43 ± 0.31^b^	18.43 ± 0.31^b^
40	29.33 ± 30.16^c^	37.10 ± 0.19^c^	35.38 ± 0.26^c^	29.99 ± 0.12^c^
80	36.59 ± 0.23^d^	56.33 ± 0.16^d^	57.10 ± 0.49^d^	45.38 ± 0.21^d^
160	47.10 ± 0.39^e^	73.48 ± 0.27^e^	69.43 ± 0.11^e^	48.43 ± 0.33^e^
320	59.33 ± 0.17^f^	87.10 ± 0.39^f^	75.38 ± 0.06^f^	59.99 ± 0.13^f^
640	76.59 ± 0.13^g^	96.33 ± 0.37^g^	87.11 ± 0.41^g^	77.38 ± 0.61^g^
IC50	646.59 ± 0.2	926.33 ± 0.16	817.10 ± 0.43	738.13 ± 0.34

Note: different superscripts indicate statistical differences within the column.

**Table 2:** Antimicrobial activity of different extract of *R. arboreum* flowers.

Extract	Bacteria	Concentrations of extract			
		25%	50%	75%	100%
LGP	*Aeromonas hydrophila*	1.37 ± 0.56^a^	4.69 ± 0.16^b^	7.13 ± 0.54^c^	9.94 ± 0.12^d^
	*Staphylococcus aureus*	4.79 ± 0.08^a^	8.81 ± 0.78^b^	12.26 ± 0.11^c^	15.13 ± 0.92^d^
	*Escherichia coli*	1.91 ± 0.11^a^	5.89 ± 0.31^b^	11.58 ± 0.68^c^	16.42 ± 0.66^d^

LGE	*Aeromonas hydrophila*	3.51 ± 0.88^a^	7.22 ± 0.27^b^	9.42 ± 0.65^c^	18.28 ± 0.94^d^
	*Staphylococcus aureus*	1.76 ± 0.19^a^	8.16 ± 0.37^b^	10.09 ± 0.56^c^	21.41 ± 0.63^d^
	*Escherichia coli*	4.75 ± 0.55^a^	9.26 ± 0.47^b^	14.28 ± 0.94^c^	17.85 ± 0.05^d^

LGA	*Aeromonas hydrophila*	2.39 ± 0.68^a^	8.33 ± 0.41^b^	12.86 ± 0.27^c^	17.16 ± 0.65^d^
	*Staphylococcus aureus*	2.85 ± 0.81^a^	7.13 ± 0.55^b^	11.21 ± 0.96^c^	19.85 ± 0.06^d^
	*Escherichia coli*	3.83 ± 0.12^a^	6.24 ± 0.49^b^	10.31 ± 0.17^c^	20.13 ± 0.93^d^

Note: different superscripts indicate statistical differences within the row.

## Data Availability

The data that support the findings of this study are available from the corresponding author upon reasonable request.
